# High molar activity [^18^F]tetrafluoroborate synthesis for sodium iodide symporter imaging by PET

**DOI:** 10.1186/s41181-022-00185-w

**Published:** 2022-12-13

**Authors:** Dmitry Soloviev, Piotr Dzien, Agata Mackintosh, Gaurav Malviya, Gavin Brown, David Lewis

**Affiliations:** 1 Cancer Research UK Beatson Institute, Garscube Estate, Switchback Road, Glasgow, G61 1BD UK; 2 School of Cancer Sciences, University of Glasgow, Garscube Estate, Switchback Road, Glasgow, G611QH UK

**Keywords:** NIS, Reporter gene imaging, Tetrafluoroborate, Fluorine-18, Positron emission tomography, Sodium iodide symporter

## Abstract

**Background:**

Sodium iodide symporter (NIS) imaging by positron emission tomography (PET) is gaining traction in nuclear medicine, with an increasing number of human studies being published using fluorine-18 radiolabelled tetrafluoroborate ([^18^F]TFB). Clinical success of any radiotracer relies heavily on its accessibility, which in turn depends on the availability of robust radiolabelling procedures providing a radiotracer in large quantities and of high radiopharmaceutical quality.

**Results:**

Here we publish an improved radiolabelling method and quality control procedures for high molar activity [^18^F]TFB. The use of ammonium hydroxide for [^18^F]fluoride elution, commercially available boron trifluoride-methanol complex dissolved in acetonitrile as precursor and removal of unreacted [^18^F]fluoride on Florisil solid-phase extraction cartridges resulted in the reliable production of [^18^F]TFB on SYNTHRA and TRACERLAB FX_FN_ automated synthesizers with radiochemical yields in excess of 30%, radiochemical purities in excess of 98% and molar activities in the range of 34–217 GBq/µmol at the end of synthesis. PET scanning of a mouse lung tumour model carrying a NIS reporter gene rendered images of high quality and improved sensitivity.

**Conclusions:**

A novel automated radiosynthesis procedure for [^18^F]tetrafluoroborate has been developed that provides the radiotracer with high molar activity, suitable for preclinical imaging of NIS reporter gene.

## Background

In this work we publish results of our efforts to develop a simple reliable automated radiolabelling procedure for [^18^F]tetrafluoroborate ([^18^F]TFB) to meet molar activity requirements for small animal reporter gene imaging of the sodium iodide symporter (NIS) (Khoshnevisan et al. 2016).

Imaging human NIS (Dohán et al. 2003) by radioactive iodide and pertechnetate has been a cornerstone of thyroid imaging in nuclear medicine (Chang 2002). Since the introduction of [^18^F]TFB into PET by Blower et al. in 2010 (Jauregui-Osoro et al. 2010)and the first successful imaging of thyroid in patients (O'Doherty et al. 2017), this tracer has gained more and more attention from the nuclear medicine community (Verburg et al. 2020)and is already proving useful in clinical applications (Dittmann et al. 2020).

Emerging roles and prospects for [^18^F]TFB in NIS imaging, both in clinical PET imaging of thyroid cancer and preclinical research using NIS as a reporter gene, have been recently reviewed (Jiang and DeGrado 2018) and confirmed the utility and promising future of this radiotracer. Human dosimetry, pharmacokinetics and biodistribution of [^18^F]TFB were studied on healthy volunteers (Jiang et al. 2017) and thyroid cancer patients (O'Doherty et al. 2017).

The future success of any PET tracer can be either enhanced or hindered by its availability, which in turn strongly depends on the availability of simple reliable high-yielding radiosynthesis procedures, providing the tracer with good radiopharmaceutical quality (Zimmermann and Chrysalium 2013).

An inherent problem related to the synthesis of fluorine-18 labelled tetrafluoroborate is its low molar activity (A_m_), ranging from 1 to 9 GBq/µmol at the End of Synthesis (EOS) (Jiang and DeGrado 2018), while for conventional fluorine-18 labelled radiotracers molar activities as high as 370–740 GBq/µmol are achievable (Sergeev et al. 2018).

Studies by Blower et al. have convincingly shown, that the amount of carrier tetrafluoroborate present in the [^18^F]TFB formulation competes with the radioactive [^18^F]TFB for the transporter, thus reducing its sensitivity in detecting tumours expressing NIS (Khoshnevisan et al. 2016; Weeks et al. 2011). Therefore, developing radiolabelling procedures yielding high molar activity [^18^F]TFB is needed, especially for preclinical gene reporter studies, where saturation of the transporter could occur at much lower mass amounts (Kung and Kung 2005).

Several [^18^F]TFB radiosynthesis procedures published to date have used different approaches, none resulting in a robust, high-yielding procedure providing [^18^F]TFB of high molar activity (Jiang and DeGrado 2018). The original radiolabelling first published by Jauregui-Osoro et al. (Jauregui-Osoro et al. 2010) used isotopic exchange reaction, yielding a 1 GBq/µmol product at the end of synthesis. Radiolabelling procedures based on nucleophilic addition of [^18^F]fluoride to different boron trifluoride substrates (Jiang et al. 2016) have proven advantageous as compared to the isotope exchange approach (Jauregui-Osoro et al. 2010).

Further improvement to the synthetic procedure published by Blower et al. made use of nucleophilic addition reaction of [^18^F]fluoride with strongly diluted commercial boron trifluoride etherate, which improved the molar activity to 5.7 ± 3.5 GBq/µmol at EOS (Khoshnevisan et al. 2016). The next improvement published by DeGrado et al. (Jiang et al. 2016) was an automated procedure making use of freshly prepared gaseous boron trifluoride on an anion-exchange support to perform nucleophilic addition, which resulted in an expedient (10 min) radiosynthesis with higher radiochemical yields (20%) and higher molar activity (9 GBq/µmol at EOS), the best result published so far.

In the same year a CRUK Cambridge Institute group (Soloviev et al. 2016) reported a manual radiolabelling procedure of the easily accessible commercial reagent boron trifluoride dihydrate, thus simplifying the synthetic procedure (20–40% overall radioactivity yield in 15 min), but the molar activity of the final preparation was surprisingly low (0.01 ± 0.04 GBq/µmol at EOS).

**Fig. 1:**
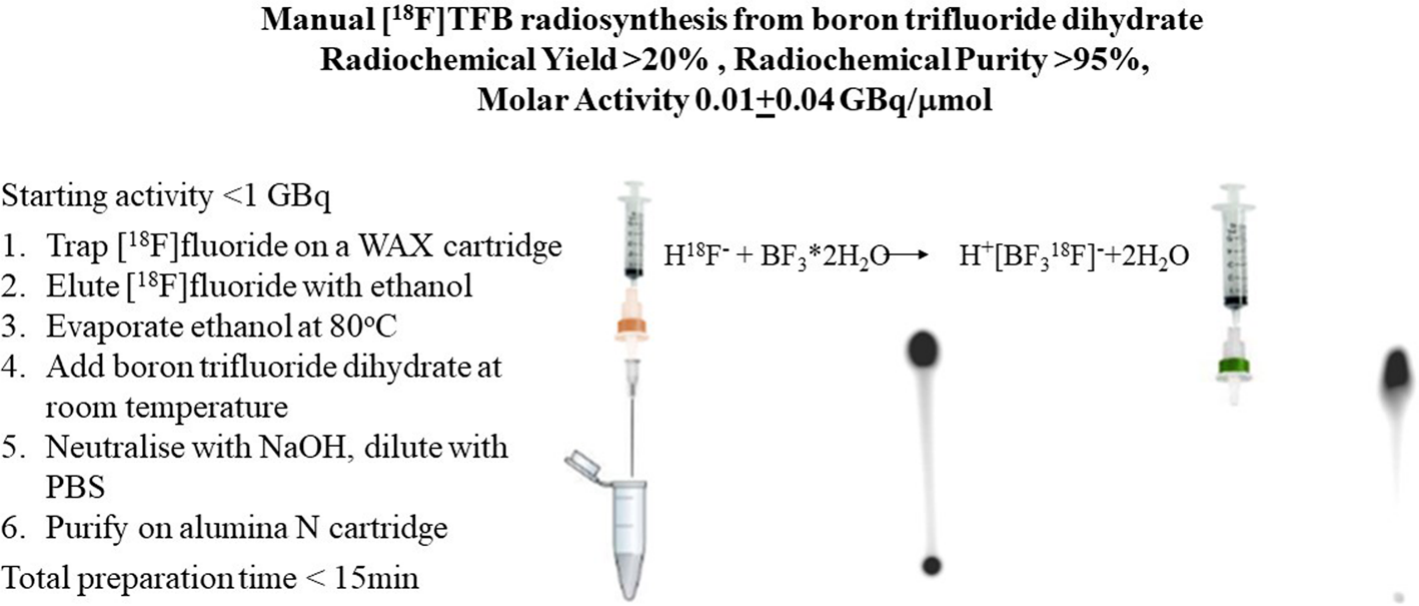
Manual radiolabelling procedure of boron trifluoride dihydrate (adapted with permission from Soloviev et al. (Soloviev et al. 2016))

Direct addition of [^18^F]fluoride to boron trifluoride dihydrate proceeds instantly at room temperature with more than 80% [^18^F]fluoride incorporation, as monitored by thin layer chromatography (Soloviev et al. 2016) (Fig.1). The fast reaction rate is predictable, given the ionic nature of the boron trifluoride dihydrate (Wamser 1951):

$${\text{BF}}_{3} *2{\text{H}}_{2} {\text{O}} \to {\text{H}}_{3} {\text{O}}^{ + } {\text{BF}}_{3} {\text{OH}}^{ - }$$

**Fig. 2:**
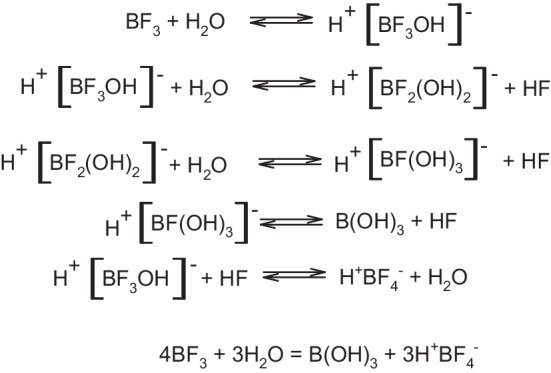
Multistep hydrolysis of boron trifluoride leading to boric acid and tetrafluoroboric acid

Low molar activity of the [^18^F]TFB prepared from boron trifluoride dihydrate (median A_m_ 0.01 ± 0.04 GBq/µmol at EOS) could not be attributed only to low starting fluoride activity (< 1GBq). The high mass of the non-radioactive tetrafluoroborate present in the final preparation could be explained by the multi-step hydrolysis of boron trifluoride upon addition of water (Wamser 1951) providing tetrafluoroborate and boric acid in the following summary equation, Fig.2:

In fact, it was shown, that the most stable species in the borate-tetrafluoroborate aqueous mixtures are boric acid and tetrafluoroborate (Grassino and Hume 1971; Katagari et al. 2006). The same hydrolysis reaction could explain all the low molar activity results published by other groups—as soon as the reaction mixture after radiolabelling is subjected to aqueous purification steps, the excess boron trifluoride reagent undergoes hydrolysis, thus increasing the mass of tetrafluoroborate in the final preparation.

Of note were low radioactivity yields achieved after purification (in the range of 20–30%) despite a short radiosynthesis time (less than 15 min) and high [^18^F]fluoride incorporation into [^18^F]TFB (Fig. 1). This observation corroborated well with other reports (Khoshnevisan et al. 2016; Jauregui-Osoro et al. 2010; Jiang et al. 2016), when low radiochemical yields, despite the high fluoride incorporation, could be attributed to the use of two large format alumina purification cartridges for fluoride removal—refer to (Khoshnevisan et al. 2016) and the "Discussion" section below.

The first obvious strategy to improve the molar activity of [^18^F]TFB would be reducing the amount of the boron trifluoride precursor to a minimum. The trade-off for reducing the precursor amount would be a reduction in radiochemical yields. In fact, all the groups developing [^18^F]TFB radiosynthesis have reported lower radiochemical yields after optimisation of their radiolabelling procedures to achieve maximum molar activity (Khoshnevisan et al. 2016; Soloviev et al. 2016). Therefore, a balance should be struck between the radiochemical yield and the amount of starting boron trifluoride reagent. There are limitations in minimising the concentrations and amounts of precursor for radiolabelling, unless the novel microdroplet chip technologies are implemented (Sergeev et al. 2018).

**Fig. 3:**
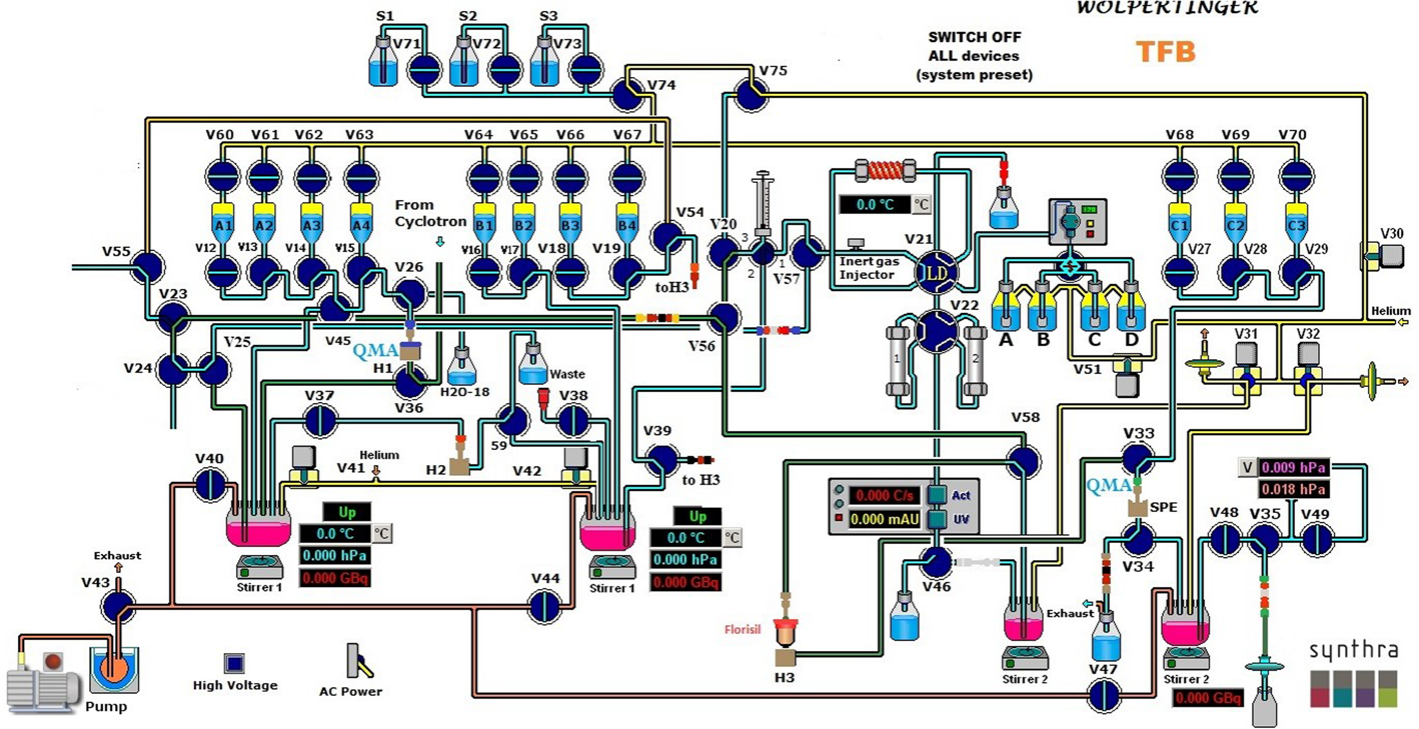
Schematic diagram of the SYNTHRA setup for [^18^F]TFB synthesis

In the absence of chemical chip technology, we present here a new radiochemical synthesis procedure for [^18^F]TFB using two automated synthesizers, namely TRACERLAB FX_FN_ and SYNTHRA (Fig. 3).

## Materials and methods

### General

Methanol, acetonitrile (HPLC grade) and ethanol (HPLC grade) were purchased from Rathburn Chemicals UK Ltd. All the reagents, ammonium tetrafluoroborate, anhydrous acetonitrile, and anhydrous methanol were purchased from Sigma Aldrich (UK). Sep-Pak Accell Plus QMA Carbonate Plus Light Cartridge (46 mg of sorbent per cartridge, 40 μm particle size), Sep-Pak alumina N Plus Light Cartridge, Oasis WAX Plus short cartridge (225 mg sorbent per cartridge, 60 μm particle size), and Sep-Pak Florisil Plus Light Cartridge were purchased from Waters UK. 0.9 % saline was purchased from Greater Glasgow & Clyde NHS Pharmacy Distribution Centre. All Sep-Pak cartridges were used without prior conditioning.

Boron trifluoride acetonitrile complex solution, 15.2–16.8% BF_3_ basis; boron trifluoride-methanol solution, ~ 10% (~ 1.3 M), for GC derivatization; boron trifluoride dihydrate, 96% and aqueous ammonium hydroxide (25%) were purchased from Sigma-Aldrich. Precursors for [^18^F]TFB radiolabelling were diluted in an anhydrous solvent to the desired concentration as described in the "Results" and Discussion" sections. To obtain 1/500 dilution 3 µL of 10% boron trifluoride methanol solution was withdrawn from the original 10 mL shipment bottle by means of the 10 µL glass GC syringe and dissolved in 1500 µL of anhydrous acetonitrile in a sealed 2mL glass HPLC sample vial.

### Radiochemistry

No-carrier-added [^18^F]fluoride was obtained through the ^18^O(p, n)^18^F nuclear reaction by irradiation of 95–97 atom % ^18^O enriched water (purchased from Sercon UK Ltd) in a niobium target chamber (2.7 mL target volume) with a 16.4 MeV proton beam on the GE Healthcare PETtrace cyclotron at the West of Scotland PET Centre. Typically, an 80 µA, 15 min target irradiation gave 50.1 GBq of [^18^F]fluoride at the end of bombardment (EOB) for use at the start of synthesis (SOS), which times were circa 10 min apart for target unloading procedures.

The fully automated synthesis of [^18^F]TFB was carried out either on a customised SYNTHRA module (Synthra GmbH, Germany) (Fig. 3) or on the TRACERLAB FX_FN_ (GE Healthcare). Different boron trifluoride precursors, solvents, solid phase extraction cartridges and varying drying step and reaction conditions were tested in the development phase, as discussed in the "Results" and "Discussion" sections.

The optimised radiolabelling procedure was conducted as follows:

Cyclotron produced, no-carrier-added [^18^F]fluoride was passed through a QMA-carbonate SepPak light cartridge. The trapped [^18^F]fluoride was eluted from the cartridge into a 2.5 mL glass reaction vessel with 200µL solution of ammonium hydroxide (25% in water) diluted in methanol 1/10 V/V.

After evaporation to dryness at 50 °C under the reduced pressure and a flow of helium for approximately 20 min, 150 µL of boron trifluoride-methanol complex (10%) diluted in anhydrous acetonitrile 1/500 V/V was added (total 0.39 µmol BF_3_ equivalent). The radiolabelling reaction was performed at 75 °C in a closed reaction vessel for 7 min. Upon completion the reaction mixture was dissolved in 4 mL of anhydrous ethanol (added in two consecutive portions) and passed through the Florisil Light, and QMA-carbonate Light Sep-Pak solid phase extraction cartridges connected in series. After rinsing QMA cartridge with 10 mL of water for injections the final product was eluted by 6 mL of isotonic saline into a sterile vial. Total duration of the synthesis was 34 min.

### Quality control

Thin Layer Chromatography: methanol mobile phase run on alumina plates (FLUKA 89071-50EA aluminium oxide on TLC-PET foils with fluorescent indicator 254 nm aluminium oxide matrix). A Mini-scan radio–thin-layer chromatography (radio-TLC) scanner from Bioscan, Inc., was used to monitor the radiochemical purity.

High Performance Liquid Chromatography: anion exchange HPLC in basic medium as described by Katagari et al. (Katagari et al. 2006) was performed on an IC-2100 (Dionex) system equipped with conductivity and radioactivity detectors connected in series; eluent − 35 mM sodium hydroxide; sample volume 25 µL; flow rate 0.4 mL/min; column—Dionex IonPac AS20 analytical 2 × 250 mm equipped with Dionex IonPac AG20 2 × 50 mm guard pre-column. Conductivity detector response was calibrated with ammonium tetrafluoroborate standard solutions to measure the carrier [^18^F]TFB concentration in the final preparation.

### PET imaging

#### Transgenic cell generation and culture

Clonal, transgenic A549-LN cell line was derived from A549 human non-small cell lung carcinoma line, as described previously (Dzien 2022).

Briefly, NIH A549 cell line was purchased from ATCC and cultured in RPMI1640 (#21,870,084) supplemented with 2mM Glutamine (#25,030,081), both purchased from ThermoFisher Scientific (Life Technologies), and 10% foetal bovine serum (Gibco). Absence of *Mycoplasma* contamination in cell culture was confirmed by regular in-house testing.

Lentiviral vector carrying Luc2-P2A-mNIS reporter gene cassette under the *PGK*promoter (Rodriguez et al. 2014; Tiscornia et al. 2006) was used for A549 line transduction, which was followed by limiting dilution sub-cloning (Dzien 2022). Transgene expression in thus generated clonal A549-LN cell line was confirmed by in vitro bioluminescence imaging (Luc2) and [^18^F]TFB uptake experiments (mNIS) (Dzien 2022) (data not shown).

#### Tumour cell transplantation

9–14-week-old NOD/NcrCrl (*Prkdc*^scid^) mice were purchased from Charles River UK. Shortly before transplantation A549-LN cells were harvested by trypsinisation and re-suspended in ice-cold PBS. To establish orthotopic tumours, a 200 µL sample of cell suspension, containing 2.5 × 10^5^ cells, was injected intravenously.

#### PET-MR imaging and data analysis

Animals were imaged 7 and 12 weeks from cell transplantation. Animals were anaesthetised with 1.0–2.5% isoflurane in 95% oxygen and a cannula was inserted into the tail vein. Animals were injected intravenously with 0.30–0.45 MBq of [^18^F]TFB per g body weight in 200–250 µL saline (0.9% NaCl) and transferred to a NanoScan PET/MRI (1T) (Mediso, Hungary). The respiration rate of the animals was monitored by a pneumatic pad for the duration of the imaging session and their body temperature was maintained by the flow of heated air. Coronal T1-weighted images, used for anatomical reference and attenuation correction, were acquired using 3D gradient-recalled echo sequence (TR 22.5 ms; TE 3.8 ms; flip angle 30°; data matrix, 256 × 256; slice thickness 0.70 mm; 48 slices). A 20-min static PET image was then acquired, starting 70 min from the injection of [^18^F]TFB.

Image reconstruction was performed using 3D Tera-Tomo software (Mediso Medical Imaging Systems, Hungary). PET scans were reconstructed using static, total-body mode with 4 iterations and 6 subsets and an energy window 400–600 keV, producing a 0.4 mm isotropic matrix. PET data were corrected for radioactivity decay, random coincidences, scatter, attenuation and dead time. Scatter and attenuation correction used the T1 3D GRE MR images. The reconstructed PET scans were co-registered with MRI scans for anatomical reference. PET/MR data were visualised using VivoQuantTM multi-modality post-processing suite (Invicro, USA).

Standardized uptake values (SUV) were calculated using:

$$\text{SUV}=\frac{\text{c} \text{i}\text{m}\text{g}}{ID/BW };$$

where c_img_ is the activity concentration (MBq/mL) derived from the image ROI, ID is the injected dose, and BW is the body weight of the animal.

## Results

**Fig. 4:**
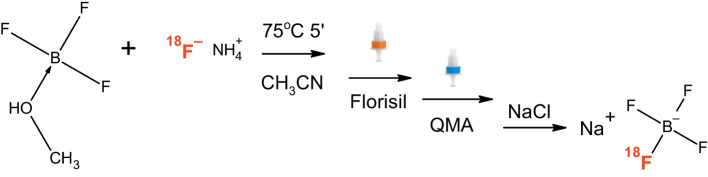
Optimised procedure for [^18^F]TFB production

We have developed a simple and reliable automated process (Fig. 4) to produce [^18^F]TFB with high molar activity (range 34–217 GBq/µmol at the end of synthesis (EOS), depending on the starting activity. The average radioactivity yield was 16.1 ± 6.0 GBq of isolated purified product at EOS starting from 51.9 ± 11.2 GBq (range 25-102.3 GBq) [^18^F]fluoride at EOB with overall process time 34 min from the moment of target download into the synthesizer (TRACERLAB FX_FN_ and SYNTHRA in our hands, Table 1). The molar activity range was 33.9-216.7 GBq/µmol at EOS, depending on starting activity, average radiochemical purity was 98.4 ± 1.5%. The process can be easily deployed on most modern radiochemistry synthesizers.

**Table 1:** Summary of the [^18^F]TFB production development studies

Synthesizer	Solvent	Dilution	Activity at EOB (STD) (GBq)	RCY (CV) (%)	RCP (STD) (%)	A_m_ (range) at EOS (GBq/µmol)	A_m_ normalised to activity at SOS (µmol^−1)^
Precursor: BF_3_*MeOH (10%)							
SYNTHRA (n = 4)	CH_3_CN	1/600	103 (21)	24 (17)	98.8 (0.7)	90 (32–168)	0.9
SYNTHRA (n = 9)*	CH_3_CN	1/500	52 (11)	41 (18)	98.4 (1.5)	78 (34–217)	1.4
SYNTHRA (n = 9)	CH_3_OH	1/100	15 (14)	29 (24)	98.5 (1.5)	19.8 (6–96)	1.32
SYNTHRA (n = 5)	CH_3_OH	1/50	65(15)	35(17)	97.6 (0.9)	15 (0.9–27)	0.23
Precursor: BF_3_*CH_3_CN (15.2–16.8%)							
SYNTHRA (n = 9)	CH_3_CN	1/250	6(5)	55 (36)	98 (3)	5.6 (2.5–23)	0.93
FXFN (n = 5)	CH_3_CN	1/300	35 (30)	50(26)	98 (1)	39.8 (2.1–99.3)	1.13
Precursor: BF_3_*2H_2_O (96%)							
FXFN (n = 2)	CH_3_OH	1/3000	48	26	99.2	54 (4.7–55.3)	0.62
Published results for comparison							
Jiang et al. (2016)			42 (2)	20 (0.7)	98	8.8 (0.6)	0.21
Khoshnevisan et al. (2016)			1.5	13.2 (5.9)		5.7 (3.5)	3.8
O'Doherty et al. (2017)			46 (6)		99.8	2.6 (1.4)	0.05
Jiang and DeGrado (2018)			15 (3)	10	96	1.1	0.07

*Conditions of choice for the optimised radiosynthesis procedure

*CV* Coefficient varation

The main features of the process include (see "Materials and methods", "Radiochemistry" sections for details):

- Elution of [^18^F]fluoride from the QMA cartridge by ammonium hydroxide.
- Radiolabelling reaction between ammonium [^18^F]fluoride and boron trifluoride (as a coordination complex with methanol or acetonitrile). The boron trifluoride precursor was a readily available commercial reagent.
- Removal of unreacted [^18^F]fluoride by Florisil solid phase extraction cartridge before the product purification.
- Trapping and purification of the product on the anion exchange solid phase extraction cartridge (QMA or strong anion exchange).
- Formulation of the final product in isotonic saline solution.

To determine optimal dilution and volume of the precursor solution we have performed a series of optimisation experiments on the SYNHTRA synthesizer with the boron trifluoride acetonitrile complex diluted in acetonitrile (Table 2). Radiochemical yields were calculated from the HPLC analysis of the crude reaction mixture. The results show that when using the SYNTHRA synthesizer the most reproducible high RCY was obtained using 150 µl of precursor solution with a 1/500 dilution. These conditions were directly transferable onto the TRACERLAB FX_FN_ platform.

**Table 2:** Summary of the optimisation experiment results

Volume (µl)	Dilution factor	RCY (%)	CV (%)
100	1/200	41.5	45.8
300	1/200	33.3	27.0
100	1/300	38.6	36.3
200	1/300	34	20.6
100	1/600	37.9	52.8
300	1/600	38.5	33.8
150	1/500	41.0	18.4

Various temperature settings in the fluoride drying step and in the reaction step has been tested with BF_3_*MeOH diluted at 1/500 with acetonitrile using optimised volume (150 µl) (Table 3). Radiochemical yields were determined on the final product after purification. Conducting solvent evaporation and drying at 60 °C and reaction in a closed vessel at 75 °C provided the highest radiochemical yield of 48%.

**Table 3:** Temperature influence

T, ^o^C drying	T, ^o^C reaction	RCY, %	n
25	90	45.7	7
60	75	48	9
80	90	34.6	23
90	90	21.8	4
95	92	19.3	14

**Fig. 5:**
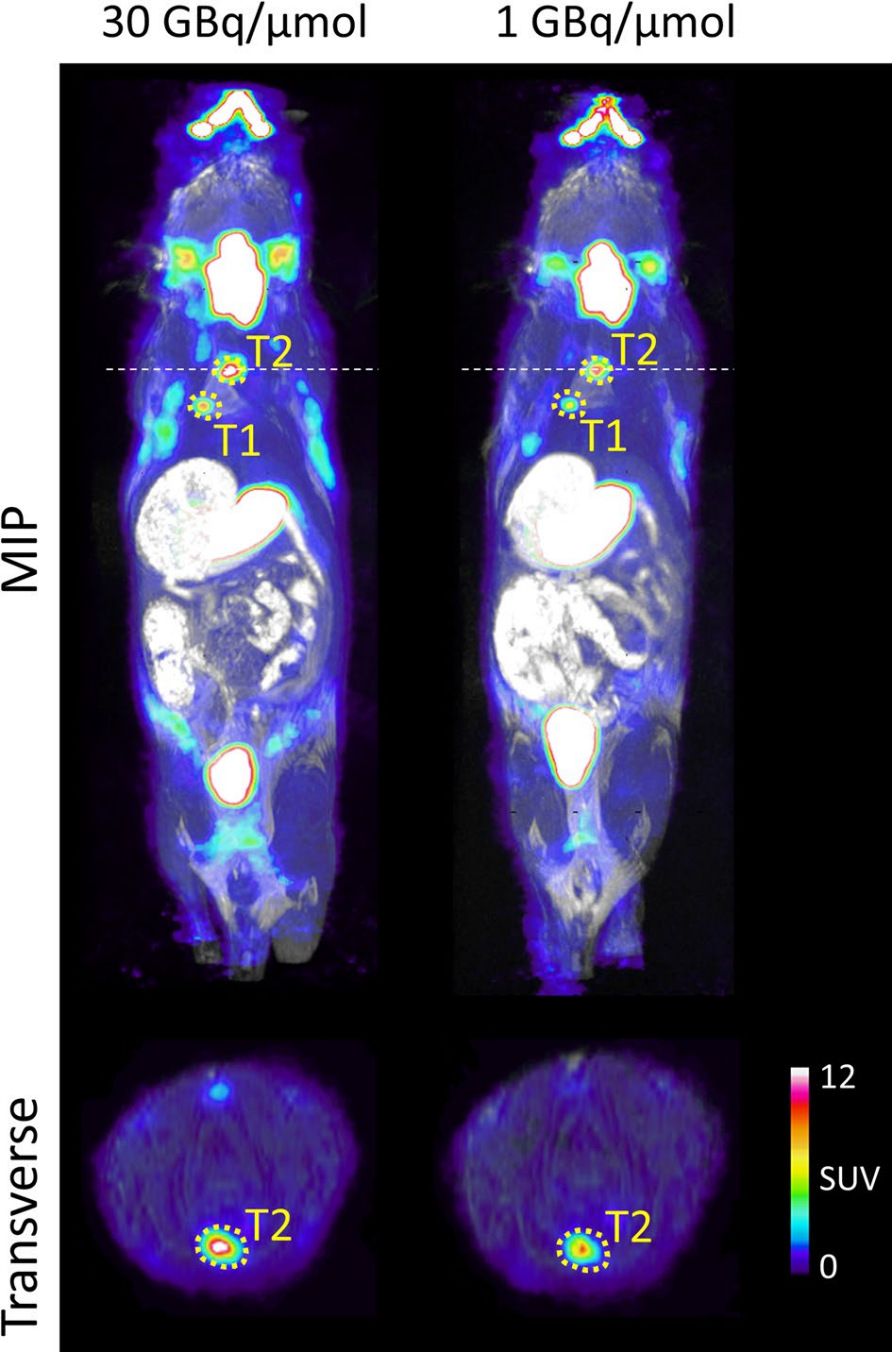
A maximum intensity projection (MIP) and transverse plane are shown on the PET/MRI of the same NIS expressing lung tumour model mouse imaged on two consecutive days, injected with 30.4 GBq/µmol (left) and 1 GBq/µmol (right) [^18^F]TFB.

PET/MRI images of a mouse harbouring NIS-expressing lung tumours were acquired on two consecutive days following injection of [^18^F]TFB either of high (30.4 GBq/µmol) or low (1 GBq/µmol) molar activity at the time of injection. Low molar activity was achieved by adding a pre-calculated carrier amount of tetrafluoroborate into the final formulation to adjust the molar activity to the desired value at the reference time (Fig. 5).

Two tumours were identified on the PET/MRI images, their SUVs and corresponding change in SUV intensity are reported in the Table 4along with SUVs of the thyroid gland (an organ expressing endogenous NIS (Jauregui-Osoro et al. 2010)).

**Table 4:** Standardised uptake values and their relative change in NIS expressing tumours and thyroid in mice injected with high (30 GBq/µmol) and low (1 GBq/µmol) molar activity

ROI	SUV, High A_m_	SUV, Low A_m_	SUV decrease (%)
Thyroid	43.8	33.8	22.9
T1	8.5	7.3	14.3
T2	15.4	10.7	30.6

## Discussion

A range of readily available commercial boron trifluoride precursors have been tested in different reaction media and employed on two different synthesizers (Table 1). There was no notable difference in terms of isolated radiochemical yields when different boron trifluoride source reagents were used.

The radiochemical yields were generally better and more reproducible when more concentrated precursor solutions were used, but the more concentrated solutions provide the final product in low molar activity. Diluting the commercial source of boron trifluoride to 1/600 by volume still provided acceptable RCY 24% (range 17.6–28.4%) with good molar activity (maximum obtained was 167.7 GBq/µmol [^18^F]TFB at EOS starting from 113.1 GBq [^18^F]fluoride at EOB).

In the optimised procedure we chose the boron trifluoride-methanol complex as a starting material mainly because it is readily available as a 10% boron trifluoride methanol solution in sealed 10 mL dark glass bottles, which facilitates the use of the reagent. For diluting the CH_3_OH*BF_3_ to the final concentration of 2.6 mM we chose acetonitrile ( 1/500 V/V dilution) mainly for the convenience of the sure-seal bottles of the anhydrous acetonitrile as supplied by Sigma-Aldrich. These conditions (see "Materials and Methods", "Radiochemistry" section for details) provided RCY 41.0% (range 30.2–56.0%) and A_m_ in the range of 33.9–216.7 GBq/µmol at EOS, depending on the starting activity.

To compare the molar activity obtained in this study with the results reported in the literature we have normalised the molar activity at EOS by the starting activity at SOS, the resulting normalised molar activity (A_mn_) expressed in µmol^−1^ allows direct comparison of the results without taking into consideration the starting activity. The best average A_mn_ in this report was 1.4 µmol^−1^ with the boron trifluoride-methanol complex dissolved in acetonitrile with 1/500 dilution (V/V). This is considerably better compared to most of the published procedures (see Table 1). The best published A_mn_ of 3.8 µmol^−1^was reported by Khoshnevisan et al. (Khoshnevisan et al. 2016). Nevertheless, we were not able to find follow-on publications from this group on large scale high molar activity [^18^F]TFB studies, probably due to the fact that the impressively high values were achieved employing laborious and moisture-sensitive serial dilutions techniques of the boron trifluoride precursor (boron trifluoride-etherate diluted in anhydrous acetonitrile to 0.8µM). Robustness of the process is of paramount importance for routine production of the tracer for clinical and pre-clinical studies.

Another means to improve the molar activity (other than diluting the precursor) could be minimising the mass of precursor in the radiolabelling by reducing the volume of the solution used. Decreasing the volume or increasing the dilution factor leads to less stable radiochemical yields, as evidenced by the coefficient of variation (CV) of the radiochemical yields (RCY) from the optimisation experiments in Table 2.

The source of fluorine-18 for radiolabelling of [^18^F]tetrafluoroborate has significant importance. Usually [^18^F]fluoride comes from the cyclotron target contaminated with various long-lived metal radionuclides and tritium (Bowden et al. 2009). To prepare [^18^F]fluoride for the next radiolabelling steps it is usually extracted from the irradiated target material by anion exchange on a solid support (QMA-carbonate cartridge). Consequently [^18^F]fluoride is displaced from an anion exchanger by eluting with another anion-containing solution (usually carbonate or bicarbonate) with the addition of counter-ion (normally potassium or tetrabutylammonium) and phase-transfer agents (usually Kryptofix 222 or tetrabutylammonium bicarbonate) thus facilitating dissolution of inorganic fluoride in organic solvents for further nucleophilic substitution reactions (He et al. 2014).

Khoshnevisan et al. have reported that no radiolabelling was observed when the customary Kryptofix 222/potassium carbonate mixture was used for fluoride elution from a QMA cartridge (Khoshnevisan et al. 2016). Instead, they proposed the use of sodium chloride—15C5 crown ether mixture. A possible explanation for this observation could be basicity of carbonate, making unfavourable formation of tetrafluoroborate anion (Katagari et al. 2006).

Ideally for the nucleophilic substitution reaction employed in [^18^F]TFB radiolabelling, fluoride in the reaction mixture should be without interfering anions. These conditions were achieved by trapping fluoride on a QMA cartridge and reacting it with boron trifluoride etherate directly on the solid support, as reported by DeGrado group (Jiang et al. 2016).

In our manual procedure (Soloviev et al. 2016) we trapped fluoride-18 from the irradiated water on the week anion exchange cartridge (WAX, Waters corp.) followed by elution with neat ethanol (i.e., no use of any ionic substrates in the eluate). This procedure performed well with nearly quantitative fluoride extraction into the ethanol solution and high incorporation of [^18^F]fluoride into the [^18^F]TFB in the manual setup. Unfortunately, it proved to be non-reproducible in the automated setting: often 40–50% loss of activity was observed either due to fluoride break-through in the trapping step, or its retention on the cartridge in the elution step. We attribute this irreproducible behaviour to the flow-rate factors governing anion exchange on the weak anion exchange resin. The process becomes less reliable than in the manual setup due to the uncontrollable high flow rates during target unloading and ethanol elution. Therefore, we turned to an alternative fluoride preparation procedure.

In this work we have successfully used aqueous ammonium hydroxide diluted in methanol to 2.5% for eluting [^18^F]fluoride from the QMA cartridge to obtain a reactive source of fluoride that is soluble in polar solvents and free from interfering ions. Evaporation of the eluate gave pure ammonium fluoride [^18^F]NH_4_F, that reacted quantitatively with boron trifluoride substrates in both methanol and acetonitrile complexation forms.

It shall be noted that excessively high temperatures, both in the fluoride drying step and in the radiolabelling reaction can dramatically reduce the radiochemical yields (Table 3), probably due to thermal decomposition of ammonium fluoride under vacuum at elevated temperatures (Chaiken et al. 1962).

In the published procedures, purification of the final product is achieved by solvent evaporation followed by formulation in physiological buffer solution and [^18^F]fluoride removal using an alumina solid phase extraction cartridge. It was noted by several researchers that a series of two or more alumina cartridges is needed to achieve the desired 95% radiochemical purity. We had the same experience in the beginning of this work when alumina was used for fluoride removal after product formulation.

Khoshnevisan and colleagues (Khoshnevisan et al. 2016) have supposed that alumina could catalyse hydrolysis of [^18^F]TFB. This is corroborated by studies of tetrafluoroborate decomposition in the presence of aluminium ions by Katagari et al. (Katagari et al. 2010). Yoshioka et al. has shown also, that TFB is effectively adsorbed on alumina doped with magnesium oxide (Yoshioka et al. 2007). In this study we also observed losses of the final product (20–30%) on the alumina cartridge and poor radiochemical purity when only one alumina cartridge was employed (data not presented).

**Fig. 6:**
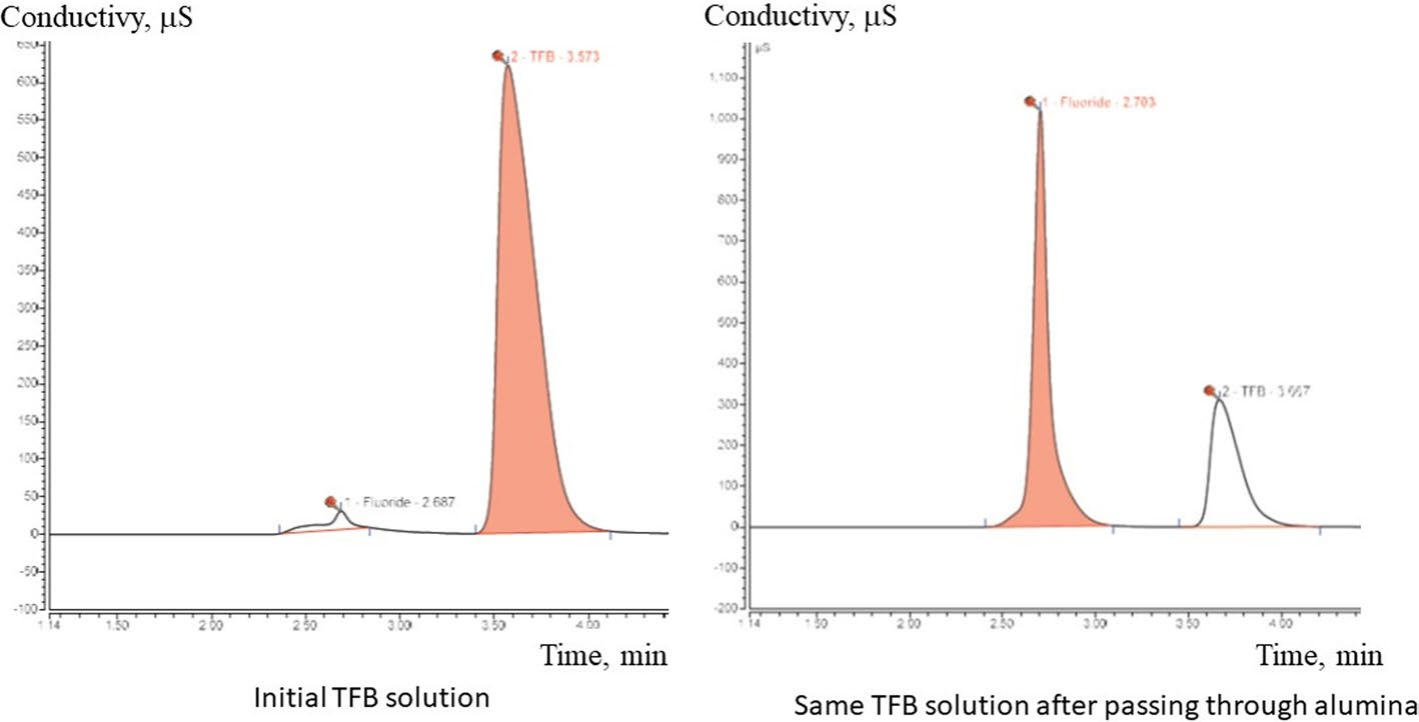
Anion-exchange HPLC trace (conductivity detector) of the TFB solution before and after passing through an alumina light SPE cartridge

A simple manual experiment consisting of passing non-radioactive TFB of known concentration through alumina and measuring fluoride and tetrafluoroborate using a conductivity detector in anion-exchange HPLC (Fig. 6) confirms TFB decomposition (rise of fluoride concentration) and its adsorption on alumina (decrease of tetrafluoroborate signal). This observation explains the reduction of radiochemical yields when alumina is employed for fluoride extraction.

**Fig. 7:**
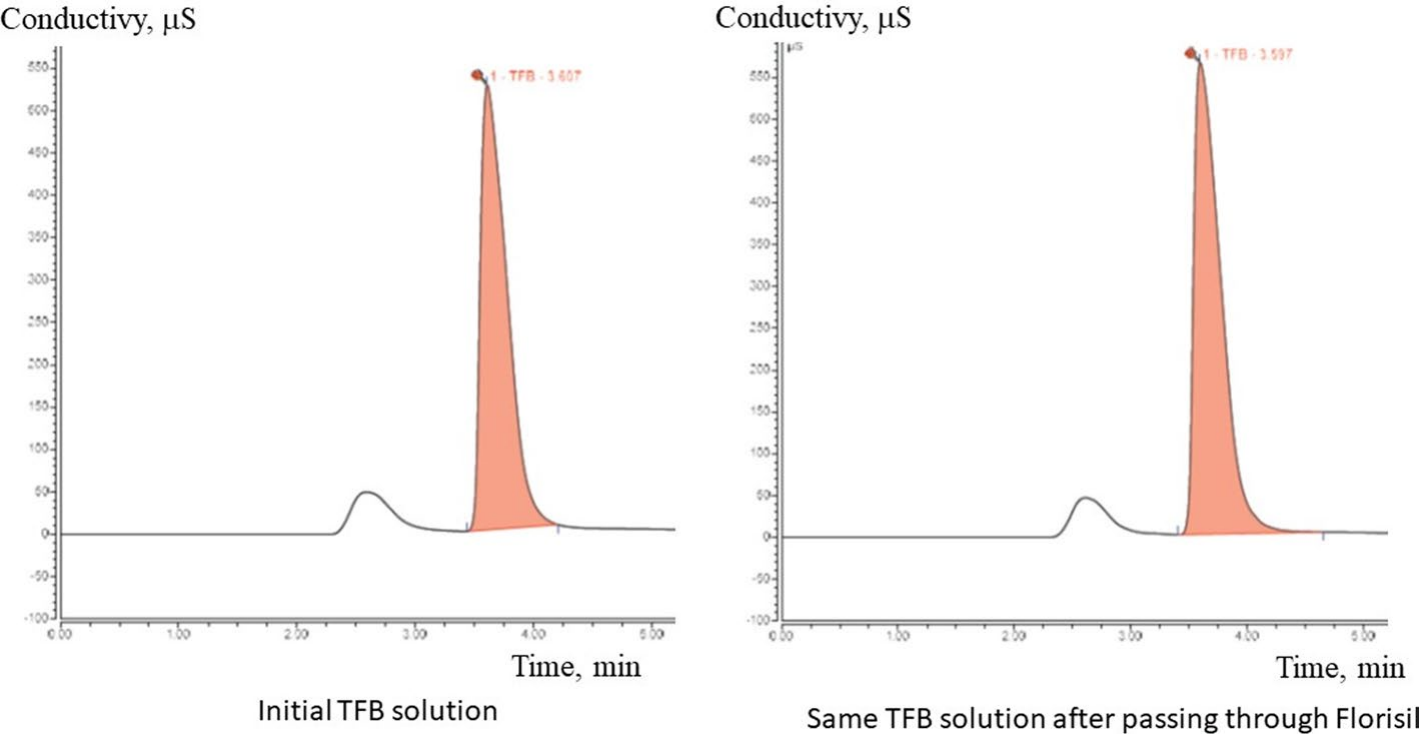
Anion-exchange HPLC trace (conductivity detector) of the TFB solution before and after passing through a Florisil light SPE cartridge

To overcome this problem, we developed an alternative purification procedure. The crude reaction mixture is dissolved in anhydrous ethanol. The mixture is passed through the Florisil and QMA solid phase extraction cartridges. Florisil is a magnesium oxide loaded silica-based adsorbent. Magnesium fluoride has the lowest solubility in water among all metal fluorides. Florisil effectively traps all the unreacted [^18^F]fluoride and does not retain [^18^F]TFB (Fig. 7). Thus, the reaction mixture dissolved in ethanol after passage through Florisil contains [^18^F]tetrafluoroborate, acetonitrile and the unreacted boron trifluoride-methanol complex. [^18^F]TFB is efficiently trapped on QMA anion exchange resin and unreacted neutral boron trifluoride passes through into the waste, thus reducing amount of carrier tetrafluoroborate. This is confirmed by the fact that while 0.39 µmol boron trifluoride equivalent is added into reaction vessel only 0.17 ± 0.07 µmol tetrafluoroborate is measured in the final formulation. After rinsing the QMA cartridge with water (10 mL) the final product is eluted with a physiologic saline solution.

## Preclinical imaging

The purpose of the imaging experiments reported here was to demonstrate suitability of the novel preparation for reporter gene imaging studies. For extensive discussion on the importance of high molar activity please refer to the work of Khoshnevisan et al. (Khoshnevisan et al. 2016).

**Fig. 8:**
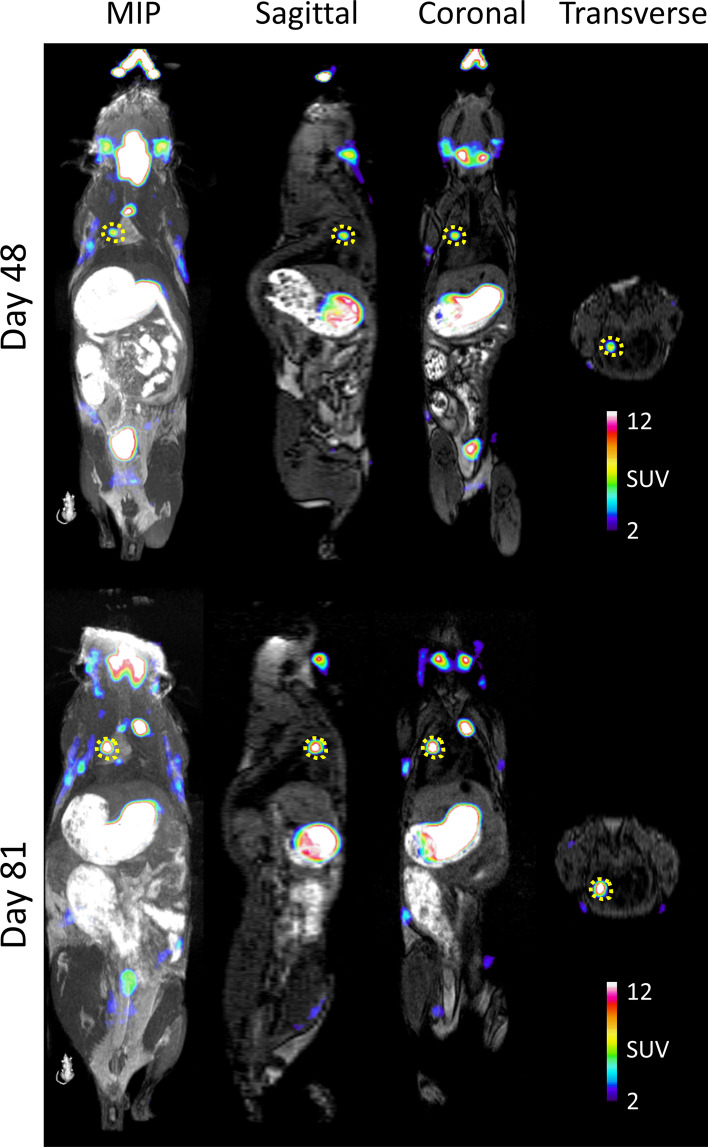
[^18^F]TFB PET imaging of NIS-expressing lung tumours over time. A small lung lesion is identified with a yellow dashed line. Orthogonal plane and a maximum intensity projection (MIP) are shown of the same mouse imaged at Day 48 and Day 81 after tumour cell transplantation

We were able to identify multiple small lung tumours with high [^18^F]TFB uptake as early as day 48 after tumour induction (Fig. 5); high tumour-to-normal lung contrast was observed. Further, we were able to follow lung tumour development longitudinally by performing repeated imaging (Fig. 8).

To demonstrate if high molar activity [^18^F]TFB preparation had effect on the imaging properties of the tracer we have compared SUVs from the NIS expressing tumours and thyroid in a mouse injected with high and low molar activity (Fig. 5; Table 4). On average 22.6% decrease was observed. We recognise limitations of drawing any conclusions from single mouse image, nevertheless our recent study (Dzien 2022) performed with the [^18^F]TFB formulation prepared according to the procedures published in this report has proven its suitability for the NIS gene reporter imaging in mice.

## Conclusion

A simplified automated radiosynthesis procedure of [^18^F]TFB for preclinical studies was developed. The procedure provides pure product (radiochemical purity better than 98%) in a reasonable radiochemical yield in excess of 30% (range 30.2–56.0%) in 34 min from the start of synthesis, which was suitable for NIS reporter gene imaging in a mouse lung tumour model. Average molar activity of the tracer was 78.5 GBq/µmol at the end of synthesis with the range 33.9–216.7 GBq/µmol, depending on the starting activity.

## Data Availability

The datasets used and/or analysed during the current study are available from the corresponding author on reasonable request.

## Abbreviations

3D: Three-dimensional
A_m_: Molar activity
A_mn_: Molar activity normalised to starting activity
EOB: End of bombardment
BW: Body weight
CV: Coefficient of variation
EOS: End of synthesis
GRE: Gradient echo
ID: Injected dose
MRI: Magnetic resonance imaging
NIS: Sodium iodide symporter
PET: Positron emission tomography
RCP: Radiochemical purity
RCY: Radiochemical yield
ROI: Region of interest
STD: Standard deviation
SOS: Start of synthesis
SUV: Standardised uptake value
TE: Echo time
TR: Repetition time
[^18^F]TFB: Tetrafluoroborate
